# Swiss chocolate and free beverages to increase the motivation for scientific work amongst residents: a prospective interventional study in a non-academic teaching hospital in Switzerland

**DOI:** 10.1186/s13063-019-3956-5

**Published:** 2020-01-13

**Authors:** Annika Rühle, Florian Oehme, Björn-Christian Link, Jürg Metzger, Henning Fischer, Michael Stickel, Dimitri E. Delagrammaticas, Reto Babst, Frank J. P. Beeres

**Affiliations:** 1 Department of General and Visceral Surgery, Cantonal Hospital of Lucerne, Spitalstrasse, 6000 Lucerne, Switzerland; 2 Department of Traumatology and Orthopaedic Surgery, Cantonal Hospital Lucerne, Spitalstrasse, 6000 Lucerne, Switzerland; 3 Emergency Department, Cantonal Hospital of Lucerne, Spitalstrasse, 6000 Lucerne, Switzerland; 4 Department of Orthopaedic Surgery, Northwestern University Feinberg School of Medicine, 676 N. Saint Clair, Chicago, IL 60611 USA

**Keywords:** Motivation for research, Increasing motivation, Improving inclusion rates

## Abstract

**Background:**

The success of a clinical trial depends on its recruitment of eligible patients; therefore, the recruitment period requires special attention. We hypothesized that with a new approach focused on continuous information and gratification, resident motivation to participate in scientific work will increase and recruitment rates will improve.

**Methods:**

Our new recruitment approach was applied to the recruitment phase of two prospective randomized trials (registered at the German Clinical Trials Register). Randomization of these trials was performed first using blinded envelopes; later a soft drink machine was used as the delivery tool of randomization as a lighthearted motivation to join scientific work and to reward the resident with free soft drinks for each recruitment. Residents were informed about the trial via a lecture and by mail. To increase interest everyone received Swiss chocolate. With a multiple choice survey we investigated the success of our actions at 6 and 12 months. Recruitment rates of the trials were evaluated and associated with the motivational approaches.

**Results:**

Our residents rated their awareness of the trials with median 9 (IQR 7;9) during the first and 8 (IQR 5;9) during the second survey and their interest in scientific work with median 7 (IQR 4;8) and 6 (IQR 5;8). The percentage of residents feeling highly motivated improved from 58% to 70%. The recruitment rates stayed stably high over time with 73% and 72% in trial 1 and 90% and 85% in trial 2; 24% of residents stated their motivation could be increased by gratifications.

**Conclusions:**

After implementation of our new recruitment approach we found positively motivated residents and high recruitment rates in the corresponding trials. We propose this procedure may help to ensure the successful initiation of clinical trials. Larger studies testing this approach are warranted.

## Background

The success of a prospective clinical trial depends not only on the feasibility of the study design to prove or disprove a thesis, but also on the ability to effectively recruit a predefined sample size in a timely manner. Recruiting study participants requires engagement of both patients and investigators [1] and can be influenced in various ways regarding, e.g., patient contact and convenience, support for recruiters, different monitoring systems, design, resources, and incentives for participants as well as clinicians [2, 3]. Well-informed clinicians are more likely to recruit patients into a trial [4], a pragmatic approach and study design is more likely to be supported by patients and clinicians [5], and a local trial manager can increase the probability of successful and timely recruitment [6].

For surgical trials, especially if targeting patients with emergency operations, participant recruitment is, in our experience, often conducted during first patient contact at the emergency department (ED). While the final clinical decision on surgical procedure and trial inclusion is made by a senior physician, first patient contact and obtaining informed consent for an operation is usually the responsibility of a young resident, who is already challenged by her or his clinical responsibilities.

As patient recruitment is a lengthy procedure, potentially eligible patients may not be informed about the possibility to participate in a suitable trial. Our experience from a non-academic hospital shows that this represents an important issue negatively influencing the recruitment process. During the preceding years several multi-center trials were carried out at our hospital with only mediocre recruitment rates. In interviews with colleagues possible reasons given were a lack of information and motivation to participate in scientific work due to a perceived lack of benefit for the resident. Given an increased emphasis on the importance of work–life balance in recent years [7], we hypothesize that the motivation to do extra work above the required clinical duties is a key factor influencing the recruitment rate, and thereby influences the success of completing prospective clinical trials, especially in hospitals without a strong scientific infrastructure.

Regarding the existing literature, a recent review inquired about possibilities for improving the recruitment activity of clinicians [8]. Only few randomized trials and observational studies exist on this matter and according to the authors a key factor is the reinforcement of potential benefits for patient and clinicians [8]. Literature shows that motivation is not sufficiently triggered by providing information alone [2]; therefore, we decided to try to increase the motivation to participate in scientific work by adding positive reinforcement and immediate gratification for the resident during the recruitment process. Our hypothesis was that recruitment rates in prospective trials can be increased by positive conditioning of well-informed residents using free chocolates, cold beverages, and acknowledgement of their support. Consequently, the primary aim of this study was to evaluate the motivation of residents and the recruitment rate of two prospective clinical trials after the implementation of a new recruitment approach. The approach consisted of distribution of free soft drinks with a vending machine that simultaneously displayed the randomization results as positive stimulation and a repetition-based information program using regular reminders via mail to improve the awareness of the trials.

## Methods

In the spring and summer of 2016, two prospective randomized clinical trials in a non-academic teaching hospital in Switzerland were initiated. The scientific substance of these two studies was unrelated to the current study and details are described elsewhere [9, 10].

To improve the success of the recruitment phase of these newly implemented trials, we proposed two approaches to increase the motivation of residents to enroll patients. First, information about the scientific and clinical background for each study was provided. Residents were informed about these during short lectures and written summaries of the trial descriptions, eligibility criteria, and goals were distributed to the residents. Secondly, compensation was given for supporting recruitment in the form of chocolate provided at the beginning and every 3 months. Soft drinks were handed out during randomization for individual participant enrollment.

The described interventions to raise residents’ motivation initially aimed only to improve the probability of a successful recruitment period of these trials. The idea to investigate the effects of these interventions on residents’ motivation using a survey was developed as the two trials went along.

### Trials

The two newly implemented trials were approved by the ethics committee, registered in the German Clinical Trials Register (trial 1 study ID DRKS00010418 on 22.06.2016, trial 2 study ID DRKS00011796 on 03.03.2017) and were performed in the surgical department. Recruitment of patients took place in the ED, where informed consent was gathered and patients were given the opportunity to participate in the respective trials after being informed about the backgrounds and schedules of the trials. All patients gave informed consent regarding the necessary operation as well as participation in the trial. Follow-up assessments took place in an outpatient clinic. Patients also were informed about and consented to further use of their data. The workload required by the resident in the emergency room for recruitment of the patients was different for each of the trials and is described below. Both trials were single-center trials.

#### Trial one: Postoperative treatment of surgically drained spontaneous soft tissue abscesses [10]

The goal of this trial is to prove the non-inferiority of more easily applicable postoperative wound care after surgically treated soft tissue abscesses, aiming to empower the patient with independent wound care and a shorter period of time off work.

The additional workload incurred by this trial was reflected in three different components. First, the resident was required to inform the patient about the ongoing trial and with consent of the patient complete the informed consent form. Second, for documentation purposes, the resident was asked to take a photo of the abscess and randomize the patient into a treatment group. Simple 1:1 randomization was used. Initially randomization was carried out by drawing a prepared envelope containing the randomization result as well as the required forms. After 6 months the envelopes, still stored at the ED, contained two coins instead of the randomization result. With the coins free soft drinks could be collected using a soft drink machine in which the cans were prepared with randomization stickers. The soft drink machine is further described below. Third, the resident needed to fill out a short questionnaire concerning basic clinical patient characteristics.

Recruitment in this trial led to an estimated additional work of 15 min for the resident in the ED. The recruitment period for this trial was planned to last 1 year.

#### Trial two: Necessity of a postoperative routine radiograph after osteosynthesis of a distal radius or ankle fracture when standardized intraoperative radiographs are taken [9]

The aim of this trial was to determine whether a postoperative radiograph is needed in addition to a standardized intraoperatively taken radiograph after osteosynthesis of distal radius or ankle fractures with the goal of reducing radiation exposure and healthcare costs.

The additional workload incurred by this trial included informing the patient of the ongoing trial, completing the informed consent, and randomization of the patient into a treatment group. Recruitment led to a smaller estimated additional workload of about 5 minutes for the resident in the ED, since the associated forms were less extensive and the trial easier to explain to the patient. The recruitment period for this trial was planned to last 2 years. Again simple 1:1 randomization was used, results were delivered using the soft drink machine previously mentioned and described below.

The estimated amounts of time of 15 and 5 min do not include the time residents spent explaining the operation and background of the trial to the patient. Patients were given as much time to think about participation as they wanted, which is not reflected in the additional workload for the resident.

### Briefing

A well-informed and motivated resident is crucial for the success of a trial, since she/he is more likely to remember to approach target patients as part of their clinical work. Furthermore, being up to date in a specific area enables her/him to inform the patient accurately, which increases the probability of inspiring the patient to participate in a trial [3].

We therefore educated residents regarding the existence, background science, and study design of both trials using the interventions listed in Table 1.

**Table 1:** Interventions to inform colleagues about the ongoing trials

What	About	Who	When
General lecture	• Study backgrounds • Eligibility criteria • Tasks at different stages	All doctors of the department	Prior to start of the trials
Personal training	• Study backgrounds • Eligibility criteria • Tasks at different stages	All residents of the department	Prior to start of the trials
Printed information	• Eligibility criteria • Tasks at different stages	All doctors of the department	Few days before the recruitment period
			Repeated every 3 months
Email	• Reminder of trial • Eligibility criteria • Tasks at different stages	Residents in charge in ED	Weekly
Email	• Progress of the trials	All residents of the department	Monthly

### Gratifications

We attempted to increase recruitment in the two trials using the following three methods.

#### Swiss chocolate

Consuming chocolate is identified to not only improve mood but also increase blood glucose level and therefore productivity [11, 12]. Before the recruitment period started for each trial, all residents and senior surgeons received chocolate gifts with printed information about the trials.

Every 2 months chocolates were given to residents and nurses at the ED to renew their motivation for both trials.

#### Barbecue

As a bigger goal, a free barbecue was promised and carried out when 80% of the patients of trial 1 had been recruited.

#### Soft drinks

**Fig. 1:**
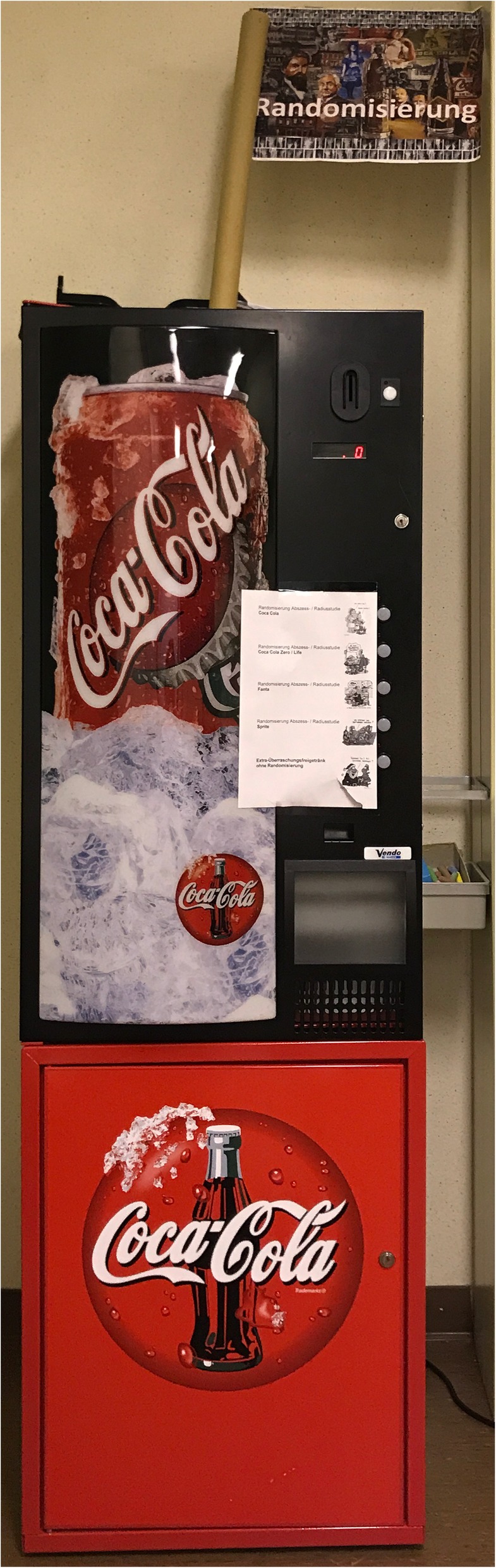
The soft drink vending machine that was used to distribute the randomization result using stickers on the cans. The machine could only be used with the coins given to the resident whilst including a patient

A vending machine was installed as an entertaining and engaging way to acquire a free soft drink (Fig. 1) in our randomization process. We used random allocation, so stickers displaying the randomization group were put on soft drink cans which were filled randomly into the soft drink machine by the authors. The amount of prepared cans correlated with the needed sample sizes for the two trials.

**Fig. 2:**
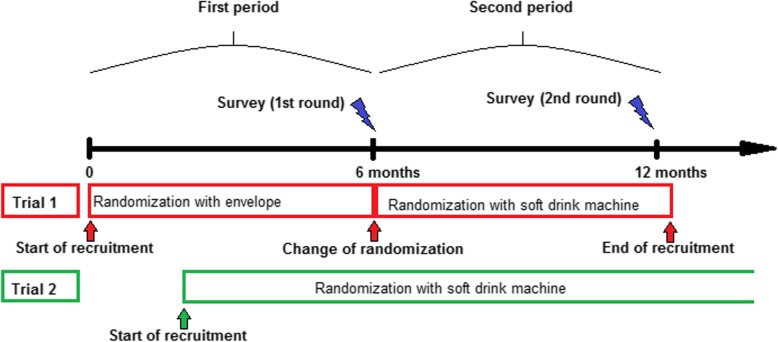
The timescale of this study as determined by the recruitment period of trial 1

For participant randomization in each trial, the resident would receive two coins for two free beverages of choice, one equipped with a sticker revealing the randomization of the patient, the second without a randomization sticker, using an extra marked tray of the vending machine. The intention of providing two beverages was to allow the resident to share with the senior doctor on call to further improve recruitment by having two motivated physicians pursuing recruitment, or at a minimum keep the resident in good graces with the senior doctor. The timeline of launching and use of the soft drink machine is displayed in Fig. 2. Given the overwhelmingly positive responses of our colleagues, we adopted the vending machine as a tool for randomization delivery in trial 1 as well. The change in randomization delivery was performed after 50% of the inclusions in trial 1 had been performed, approximately 6 months after the beginning of the trial (Fig. 2). We regarded this change in randomization delivery tool as an intervention with the intention to raise the motivation for recruiting patients into trial 1.

### Survey

**Fig. 3:**
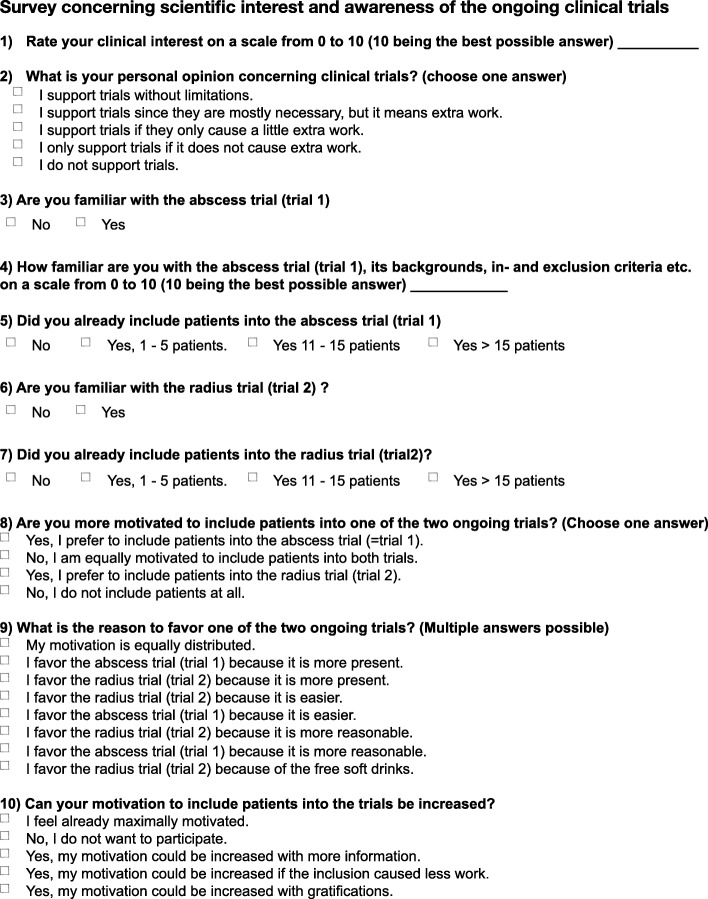
Survey that was sent to residents midway through trial 1 and at the end of trial 1 asking about their motivation, the ongoing trials, and potential influences on their motivation. Question 10 was altered during the second period: in the first survey residents were asked if they believed their motivation could be enhance externally and in the second survey residents were asked if their motivation was enhanced by better information, the given information, or their perception of the associated work

We conducted an online survey to investigate the effect of the gratification methods on the motivation for additional scientific work amongst residents and the awareness of the current ongoing trials. The survey is shown in Fig. 3 and was created using the free online tool available at https://de.surveymonkey.com. The questions cover the scientific interest of the residents in general, personal opinion regarding clinical trials, as well as familiarity with the trials (e.g., eligibility criteria) and the amount of included patients. The third part of the survey dealt with the residents’ motivation and the possibility of increasing it. The survey was sent to all residents in contact with the target population. Residents were informed about the background of the survey by cover letter.

The aim of the survey was to evaluate if the use of the soft drink vending machine was able to balance the higher amount of work required for recruitment in trial 1 compared to trial 2. A second aim was to evaluate the effects of the motivational process with regular information, chocolate, acknowledgement, and soft drinks on the inclusion rates of the two trials. To answer the first question, we chose to compare two periods of time: the first period comprises the first 6 months of trial 1 where randomization was delivered with blinded envelopes; the second period comprises the following 6 months during which the vending machine was used (Fig. 2).

In our hospital, residents regularly rotate between the ward and ED, where recruitment of the patients took place. The first survey was sent out to all residents who worked in the ED during the first period; these residents were also contacted for the second survey since they still worked either full time or night shifts in the ED. Additionally, a new cohort of residents working in the ED during the second period was asked to complete the second survey as well, leading to a bigger cohort being asked to complete the second survey. The survey was anonymous, so no definitive links between the two rounds can be made.

### Calculations

Recruitment rates of all eligible patients were calculated for the two 6-month periods for each trial (Fig. 2). For calculation of the recruitment rate the number of included patients from the cohort of eligible patients (meeting all inclusion criteria and none of the exclusion criteria) was counted. Excluded patients and those who non-consented were not counted as eligible since our interventions did not target the motivation of the patients but only the motivation of the residents to participate in scientific work.

### Statistics

Statistical analysis of the survey results was planned and carried out using SPSS version 23 (IBM Corp, Armonk, NY, USA) after completion of the survey.

Basic descriptive data were calculated as median and interquartile range (IQR). Tests for significance were chosen depending on the data characteristics (Mann–Whitney U-test, *t*-test, Chi-square test or Fisher’s exact test); a two-sided *p* value of < 0.05 was considered as a threshold of statistical significance. Comparative analyses were not adjusted for multiple testing and in these cases the displayed *p* value is of an exploratory nature.

### Reporting

Reporting of our survey results follows the guidelines for reporting survey research described by Kelley et al. [13].

## Results

### How many residents participated?

The residents response rate was 70% (19/27) in the first and 55% (23/42) in the second round of the survey.

The median response amongst residents identifying a basic interest in clinical scientific work (scale of 0–10) was 7 (IQR 4–8) in the first survey and 6 (IQR 5–8) in the second (*p* = 0.87). The multiple choice question (Question 2, Fig. 3) concerning the residents opinion of clinical trials showed that all residents were supporting clinical trials.

Two questions were designed to investigate if residents were familiar with the respective trials to revise the information process. All but two residents stated they were familiar with both trials, eligibility criteria, and the necessary procedures to include a patient. The two residents unfamiliar with the trials revealed themselves to be new colleagues that started after the implementation period.

Missing data of 30% during the first and 45% during the second survey have to be reported. These missing data can be partly explained since colleagues not yet in contact with the trials did not complete the survey, but of course it has to be considered that less scientifically motivated colleagues might have chosen not to complete the survey.

### Were the trials acknowledged by the residents?

**Fig. 4:**
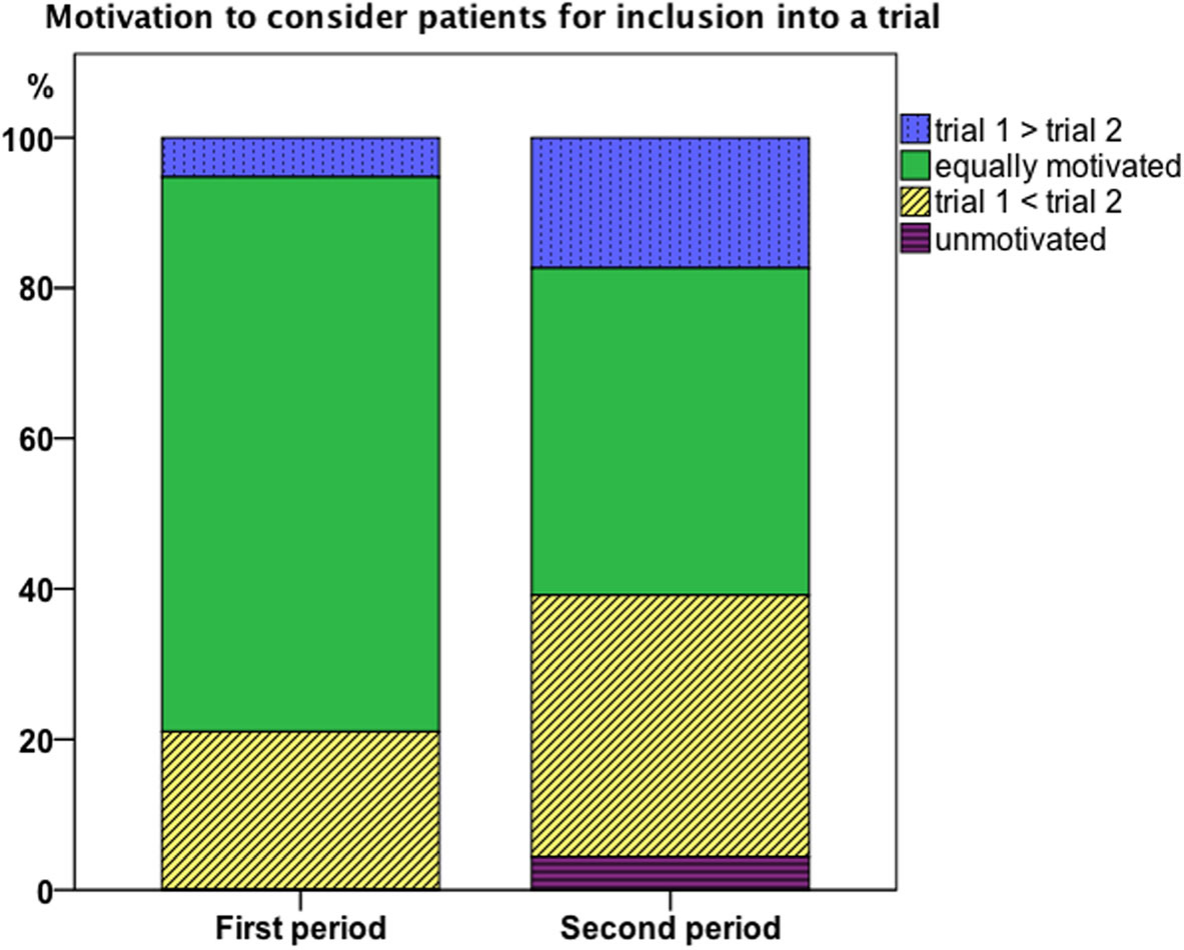
Results of the surveys concerning residents’ motivation to consider a patient for inclusion in a trial and if there were preferences toward one of the trials since trial 2 was less work for the residents

The motivation of residents to recruit patients into the trials during the first and second rounds of the survey are presented in Fig. 4. A preference for one of the two trials based on the required work and compensation were revealed during each round: trial 1 was favored by only 5% during the first survey with an increase to 17% during the second survey; trial 2 was preferred by 21% and 35% during the first and second rounds, respectively. A high amount were equally motivated, with 74% in the first and 44% in the second round of the survey. Only 4% stated they were not motivated at all during the second round.

Residents were asked for their reasons for favoring one of the two trials. Reasons for preferring one of the two trials during the first and second rounds of surveys were better establishment of a trial (meaning easier to perform for the resident) in 17% (3/18) vs 18% (4/22), easier implementation with less work in 6% (1/18) vs 32% (7/22), and a study background that was easier to understand for the resident in 22% (4/18) vs 5% (1/32). No resident stated that the soft drinks were a reason to prefer trial 2 over 1 during the first survey. During the second survey in both trials free soft drinks were given, so this was not applicable any more.

### How motivated were the residents to do scientific work?

The last question of the survey dealt with the subjective assessment of motivation. Residents had to choose in a multiple choice question asking whether they already felt highly motivated, did not want to participate in the trials at all and therefore motivation was no issue for them, or if motivation could be enhanced with better information, less work, or gratifications. Results are shown Table 2. In the beginning, 58% (11/19) felt highly motivated, which increased to 70% (16/23) after another 6 months, whilst the percentage of residents with no motivation vanished, potentially showing positive effects of the interventions. Regarding the potential factors to increase the motivation, 37% (7/19) stated that their motivation would be increased if the associated work load was smaller, which is not a new finding. Interestingly, this proportion dropped to only 30% (7/23) while the workload caused by the recruitment process remained stable since no alterations concerning this matter were made. Initially no resident thought that gratification would influence their motivation but, during the second survey, after increasing reinforcement by universal use of the soft drink tokens, > 20% of residents acknowledged the positive effects of gratifications.

**Table 2:** Can motivation to assess patients for inclusion in the trials be increased?

			Survey Round		Total
			1	2	
Can motivation be increased?	Motivation already maximized	N	11	16	27
		%	57.9%	69.6%	
	No interest in trials	N	1	0	1
		%	5.3%	0.0%	
	Increase with better information	N	1	2	3
		%	5.3%	8.7%	
	Increase with less work	N	7	7	14
		%	36.8%	30.4%	
	Increase with gratification	N	0	5	5
		%	0.0%	21.7%	
	Participants	N	19	23	42

Results of a multiple choice question: *N* equals the number of participants giving that answer; Percentage refers to the round of the survey

### Did the trials run according to plan?

The inclusion period of trial 1 lasted 13 months, whilst trial 2 took the planned 24 months to complete the recruitment period.

The recruitment rate for trial 2 (soft drink machine during entire study period) in the first vs second period was 90% (72/77) vs 85% (130/150) (*p* = 0.12). Recruitment in trial 1 (soft drink machine for second half of study only) was 73% (90/123) vs 72% (63/87) (*p* = 0.90) during the first and second periods.

## Discussion

The existing literature concerning influencing the recruitment period of trials focuses on trial design, material design, patient contact, and participant influence [2, 3]. Only few studies have investigated the recruiters. A recent review [8] concludes a key factor is the reinforcement of potential benefits for patient and clinician but admits that the quality for evidence is low and there is a gap regarding effective strategies aimed at recruiters. Working in a non-university hospital we found motivation to do extra work for science the key factor limiting the success of clinical trials. We aimed to ameliorate the recruitment period by motivating not intrinsically science-driven residents by positive reinforcement with immediate and subsequent gratification and regular information. The effect of the predefined interventions was measured using inclusion rate and adherence to the time lines of two randomized controlled trials carried out during the observation period as well as by a specialized survey that was answered by the residents of the department.

The results of the survey showed that the residents were conscious of trials and had a firm understanding of the scientific background of both prospective trials. This was problematic in earlier trials where residents were often not aware of the possibility of considering a patient for a running trial. Regarding these trials, our introduction strategy and regular information seem to have been successful in maintaining awareness of the trials.

An often described limitation of study completion is an extension of recruitment phase due to declining inclusion rate over time [6]. A review from 2011 [8] stated that two-thirds of trials fail to pass 80% of their recruitment target. Regarding our data, we found our trials to be nearly on schedule. In trial 1, the inclusion period was completed with only 1 month delay (13 instead of 12 months); in trial 2, the inclusion period was completed as planned after 24 months.

Recruitment rates found in the literature may vary depending on the kind of trial and are often not even reported in the literature. Rates that are reported, although not very recent data, range from 3 to 69%, but in surgical trials it is thought that the rate of included vs eligible patients is less than 50% [14]. Regarding this, our recruitment rates can be counted as quite high with 73 vs 72% in trial 1 and 90 vs 85% in trial 2 during the two rounds.

Naturally, several explanations can be found for these good results, for example, the highly motivated investigators or the simple infrastructure of a smaller hospital. But it is our belief that these stable and high recruitment rates are partly due to the fact that the randomization delivery tool is motivating the residents. However, further studies on this subject are needed for verification.

Regarding the motivation of the residents we found during the first survey a rate of 58% of residents already felt highly motivated, which is a subjective finding. After general implementation of the vending machine distribution this rate increased to 70%. Correspondingly, the rate of residents who felt it was possibile to be further motivated decreased from 37% to 30%. We also investigated possible reasons for further motivation and residents stated a smaller work load associated with the trial would motivate them, which is obviously not a new finding. Interestingly, initially nearly half of the residents identified the associated work to be of relevance (44%); after the intervention with the generalized use of the vending machine with free soft drinks to ease the inclusion process, only a third (33%) of residents found the workload important, while the actual workload for trial recruitment was stable. This possibly reflects the balancing effect of gratifications. What we also found was that, after generalized use of the vending machine, the percentage of residents acknowledging the motivating effects of gratification rose from 0 to 20%.

We also asked the residents which of the two trials they preferred. The two trials were different regarding the complexity for the patient, meaning trial 1 was harder to explain to the patient and took more time to explain. Also, the associated forms that needed to be completed were considerably more time consuming for trial 1. Since the literature [2, 3] as well as our residents stated that an easier to conduct trial that takes the resident less time is preferred to one that takes more time, we wondered if it would be possible to lessen the negative effect of more work with gratifications. Regarding our data, trial 2, the trial with almost no work for the resident that used the soft drink machine from the beginning, represented the perfect combination: less work and direct gratification. So it is not hard to believe that 21% of the residents clearly preferred trial 2 during the first survey. Over time this increased to 35%. Interestingly, although trial 1 causes substantially more work for the resident (15 vs 5 min) with the use of the soft drink machine we found more than a threefold increase (5 to 17%) in preference. Interestingly, when asked directly if the free soft drink was a reason to prefer trial 2 over 1, when the free drinks were only available in trial 2, no resident agreed. But since during the first survey the possible influence of gratifications on their motivation was denied by all residents and over time over 20% of residents acknowledged the motivating effects of the gratifications, it might be that residents only became aware of the soft drink as a motivating factor over time.

Although the data are not strong enough to draw firm conclusions, this study suggests that positive stimulation increases the willingness and motivation to participate in clinical trial recruitment regardless of the workload.

This study does have some limitations. With no baseline recruitment rate prior to the motivation and information interventions we cannot prove that the good rates are due only to, e.g., the vending machine as a fun randomization delivery method with immediate gratification. As the survey was anonymized, no paired controls were possible.

Since this is not a randomized controlled trial, no definite proof can be found regarding our data. Furthermore, we have to acknowledge a quite high rate of missing data regarding our surveys. One reason for the missing data may be that residents received the survey but did not interact with trial patients during that time (e.g., due to their main work in the operation room during these months or in wards other than the ED) and therefore chose not to answer. But there is also the possibility of biased data when unmotivated residents chose not to answer. It should be mentioned that, by influencing the possible recruiters with gratifications, it is possible that patients are pressured to participate in a trial. These patients later might discontinue the trial due to a lack of an independent drive to participate and therefore bias the results of the trial. Also ineligible patients might be included to achieve the gratification, which may interfere with the study protocol and further bias the results. Finally, it has to be discussed whether it is ethical to pursue a clinician to participate in scientific work. Referring to existing literature on this topic [15], we think that the amount and value of used gratifications in this study are small enough to be acceptable. Transferability to other hospitals or multi-center trials might be another limitation as the labeling and equipping of the vending machine is time consuming and a more complex randomization pattern might be hard to achieve with it. Of course, regular information can be extended via mail to a bigger cohort of people, but personal contact with residents could be difficult in a larger cohort, and we believe this to also be an important point in increasing motivation.

## Conclusions

This trial presents a new, innovative motivation approach to improve the recruitment period for prospective clinical trials. To our knowledge, no study has focused on these positive motivation methods. Based on the high and stable recruitment rates and the adherence to the planned time lines of two prospective randomized-controlled trials and the survey results, we believe that, with regular reminders, thoroughly providing background information, offering simple small gratifications, and spicing up the randomization delivery process with a soft drink machine, the motivation of residents to participate in scientific work can be increased. Further studies are required to back up our thesis.

## Data Availability

Supporting data are available upon request to the corresponding author.
